# Digital mHealth and Virtual Care Use During COVID-19 in 4 Countries: Rapid Landscape Review

**DOI:** 10.2196/26041

**Published:** 2022-11-30

**Authors:** Alison Müller, Alessandro Cau, Semakula Muhammed, Osman Abdullahi, Andrew Hayward, Sabin Nsanzimana, Richard Lester

**Affiliations:** 1 Division of Infectious Diseases Department of Medicine University of British Columbia Vancouver, BC Canada; 2 Institute of Diseases Prevention and Control Rwanda Biomedical Centre Kigali Rwanda; 3 Department of Public Health School of Health and Human Sciences Pwani University Kilifi Kenya; 4 Department of Epidemiology & Public Health University College London London United Kingdom; 5 University Teaching Hospital Butare Rwanda

**Keywords:** COVID-19, virtual care, public health, mHealth, contact tracing, telehealth, Canada, United Kingdom, Kenya, Rwanda, global health, apps

## Abstract

**Background:**

As a result of the COVID-19 pandemic, providing health care while maintaining social distancing has resulted in the need to provide care remotely, support quarantined or isolated individuals, monitor infected individuals and their close contacts, as well as disseminate accurate information regarding COVID-19 to the public. This has led to an unprecedented rapid expansion of digital tools to provide digitized virtual care globally, especially mobile phone–facilitated health interventions, called mHealth. To help keep abreast of different mHealth and virtual care technologies being used internationally to facilitate patient care and public health during the COVID-19 pandemic, we carried out a rapid investigation of solutions being deployed and considered in 4 countries.

**Objective:**

The aim of this paper was to describe mHealth and the digital and contact tracing technologies being used in the health care management of the COVID-19 pandemic among 2 high-income and 2 low-middle income countries.

**Methods:**

We compared virtual care interventions used for COVID-19 management among 2 high-income countries (the United Kingdom and Canada) and 2 low-middle income (Kenya and Rwanda) countries. We focused on interventions used to facilitate patient care and public health. Information regarding specific virtual care technologies was procured from a variety of resources including gray literature, government and health organization websites, and coauthors’ personal experiences as implementers of COVID-19 virtual care strategies. Search engine queries were performed to find health information that would be easily accessible to the general public, with keywords including “COVID-19,” “contact-tracing,” “tool-kit,” “telehealth,” and “virtual care,” in conjunction with corresponding national health authorities.

**Results:**

We identified a variety of technologies in Canada, the United Kingdom, Rwanda, and Kenya being used for patient care and public health. These countries are using both video and text message–based platforms to facilitate communication with health care providers (eg, WelTel and Zoom). Nationally developed contact tracing apps are provided free to the public, with most of them using Bluetooth-based technology. We identified that often multiple complimentary technologies are being utilized for different aspects of patient care and public health with the common purpose to disseminate information safely. There was a negligible difference among the types of technologies used in both high-income and low-middle income countries, although the latter implemented virtual care interventions earlier during the pandemic’s first wave, which may account for their effective response.

**Conclusions:**

Virtual care and mHealth technologies have evolved rapidly as a tool for health care support for both patient care and public health. It is evident that, on an international level, a variety of mHealth and virtual care interventions, often in combination, are required to be able to address patient care and public health concerns during the COVID-19 pandemic, independent of a country’s economic standing.

## Introduction

### Background

Pandemics pose a considerable threat to global health security and place an enormous strain on health care systems. The current COVID-19 pandemic has already claimed over 2.5 million lives, with over 112 million reported cases as of February 26, 2020 [1]. Delivering care remotely, supporting individuals on home isolation, monitoring infected individuals and their close contacts, and disseminating accurate information regarding COVID-19 to the public have been major challenges [2]. Although virtual care technologies have been used in a variety of health care settings prior to COVID-19, their utilization has significantly increased as they provide solutions to address the many challenges that result from maintaining physical distancing while providing essential health care. Virtual care is a rapidly growing area that is well-positioned to alleviate many issues within various health care systems including lengthy wait times, overcrowded emergency departments, and avoidable hospital readmittance [3-5]. Due to the COVID-19’s ease of transmissibility, self-quarantining and physical distancing measures have been implemented worldwide to mitigate its spread, in addition to border closures and significantly limiting the sizes of social gatherings.

In this landscape review—discussing the technology developed and used for COVID-19 management in Canada, the United Kingdom, Rwanda, and Kenya—we define virtual care as the remote delivery of health care using technology. This includes technology that facilitates video communication and text messaging between patients and their health care providers (HCPs). This broad category encompasses eHealth, telehealth, and clinical decision support tools in the field. Virtual care tools may provide care for patients from the time that they decide to access the health care system up until the end of their care experience, facilitating HCP-patient interactions and care throughout the entirety of the patient journey with minimal in-person interactions. Thus, virtual care strategies may improve access to care and quality of care, reduce health care costs, and empower patients to care for themselves while providing a medium through which they can comfortably request reliable information or advice [6]. An article released by the Canadian Medical Association has found that patients are overwhelmingly satisfied with their virtual health care during the COVID-19 pandemic [7]. At the time of publication, evidence was unavailable regarding perceptions of general virtual health care in other countries discussed in this paper.

mHealth has been defined by the World Health Organization as “the use of mobile and wireless technologies to support the achievement of health objectives” [8]. It encompasses any strategy that utilizes mobile wireless technology to deliver health care, including health and wellness apps in addition to digital wearable devices. Health care delivery through phone or video, also known as telehealth, can be classified into virtual visits and remote patient monitoring. Virtual visits, usually referred to as telemedicine, are online real time interactions between care providers and patients through a virtual care platform, a videoconferencing service, an app, or over the phone. Remote patient monitoring is the remote monitoring and collection of patients’ health data including vital signs and glucose levels depending on which specialized devices the patients are using. The current COVID-19 health crisis is an important opportunity to provide insight into the implementation, utilization, and efficacy of virtual care approaches during pandemics.

In this paper, we explore the current virtual care strategies for both patient care and case contact tracing in 4 focus countries: Canada, the United Kingdom, Rwanda, and Kenya. These countries were selected because our research group received an urgent Canadian federal research funding to deploy and study an mHealth intervention associated with our research group and established partners in those countries related to other pandemics (HIV and tuberculosis).

### Purpose

The purpose of this review was to outline and summarize the landscape of mHealth and virtual care in 4 countries (2 low-middle income and 2 high income) that have been purported to be used or enhanced specifically due to the COVID-19 pandemic and to serve as examples from diverse regions: Canada, the United Kingdom, Rwanda, and Kenya.

## Methods

The information for this landscape review was procured by accessing a variety of resources including peer-reviewed literature, gray literature (such as press releases), government and health organization websites, and firsthand experiences of this article’s authors and collaborators in Canada, the United Kingdom, Rwanda, and Kenya. We evaluated 2 high-income countries, classified by the World Bank as having a gross national income of more than US $12,535 per capita (Canada and the United Kingdom), and 2 lower-middle income countries, classified as having a gross national income of less than US $4045 per capita (Rwanda and Kenya) [9]. We focused on presenting rapidly published information and information procured from our collaborators to ensure that the information presented in this landscape review was up-to-date. It should be noted that there is extremely limited primary scientific literature regarding the development, implementation, and effectiveness of rapidly developed and deployed virtual care and public health interventions in the nations’ efforts to quell the spread of COVID-19 [10]. The authors contributed additional information regarding their country’s specific COVID-19 virtual care and public health measures, many of which were personally involved in implementing them. Communication with the authors and collaborators occurred via a combination of emailing, WhatsApp messaging, and videoconferencing using Zoom. To find press releases, information about mobile apps, and commentaries pertaining to COVID-19 technologies, search engines (predominantly Google) were used. Additional information regarding specific apps were found on their supporting app stores (eg, Google Play). Search engine queries were performed to find health information that would be easily accessible to the general public, with keywords including “COVID-19,” “contact-tracing,” “tool-kit,” “telehealth,” and “virtual care,” in conjunction with the corresponding national health authorities. We further focused on provincial health authorities in Canada only because of the diversity of COVID-19 measures undertaken by each province. The United Kingdom, Kenya, and Rwanda had more cohesive national development and implementation of interventions, so separation by regions was not necessary. We focused on procuring information pertaining to mHealth and virtual care technologies being used for both patient care and public health in the COVID-19 response. This includes interventions used for patient communication, appointment making, information platforms, and case contact tracing, among others. We understand that this method will invariably leave gaps, especially as information surrounding COVID-19 mHealth resources continues to evolve as the pandemic continues. We therefore encourage readers to seek additional resources, primary and secondary, and to contact our team regarding any errors, omissions, or suggestions to consider.

## Results

### Current Virtual Care Strategies

#### Canada

In Canada, several virtual care platforms with videoconferencing capabilities have existed and new ones emerged to assist physicians seeing patients remotely with some platforms that are offering their technology for free to assist with patient care during the COVID-19 pandemic. In Canadian provinces such as Nova Scotia and Prince Edward Island, health care websites highly recommend using Zoom for health care while other provincial health care sites do not specify a platform. The list of virtual care technologies is updated regularly with new platforms and features being developed as the COVID-19 situation evolves. In each province, virtual care “toolkits” have been developed to assist physicians when providing care for patients remotely (Multimedia Appendix 1). These can be accessed from provincial health authority websites easily found using internet search engines.

The federal government developed a nationwide contact tracing app called COVID Alert. It was developed in partnership with Shopify, Blackberry, and the Canadian Digital Service and was launched on July 31, 2020 [11]. It is a free app using Bluetooth technology, which uses Google and Apple’s exposure notification application programming interface to notify an individual who is in proximity of one who has self-identified as being COVID-positive on the app. The app specifications indicate that it does not use GPS technology to track the user’s location. The specifications also indicate that COVID Alert does not access private phone information, including the phone’s contacts, or access to health information of the user or other nearby users. As of October 19, the app has been downloaded 4.7 million times, which comprises approximately 16% of the Canadian population [12]. COVID Alert can also be used to report a COVID-19 diagnosis in most provinces, excluding British Columbia, Alberta, Nunavut, and the Yukon and Northwest Territories. In Alberta, ABTraceTogether, another Bluetooth-based “exposure notification app” for digital case contact tracing was released on May 2, 2020. This app also allows COVID-19–positive patients in Alberta to provide Alberta Health Services access to facilitate contact tracing [13]. An exposure notification app by the artificial intelligence group Mila in Quebec was released in May, which features additional information such as alerting potentially infected users and providing relevant, personalized recommendations to improve the understanding of infection risks [14]. These preliminary platforms have yet to demonstrate their superiority to traditional case contact tracing strategies when it comes to being effective while maintaining anonymity. Another app available for download by the Canadian public is the Canada COVID-19 app, powered by the Canadian company Thrive Health. It allows the user to track symptoms and contains a self-assessment tool, in addition to providing timely updates with important news and updates from Canada’s Ministry of Health.

The Government of Canada has also provided a variety COVID-19 resources to assist in evidence-based decision-making on the Canadian Institutes of Health Research website [15]. Numerous Canadian universities have synthesized COVID-19 evidence in the efforts to support policy and decision-making. McMaster University has COVID-END, which provides resources for decision-makers—researchers, presentations and products, as well as working groups. The 7 working groups, made up of international experts, focus on different aspects of COVID-19 knowledge translation: scoping, engaging, digitizing, synthesizing, recommending, packaging, and sustaining [16]. Ryerson University has developed a COVID-19 misinformation site to consolidate many internet resources focused on monitoring COVID-19 misinformation. They provide tools for reporting misinformation, fact-checking resources, and a misinformation and bots-watching dashboards, as well as a slideshow presentation debunking common COVID-19 claims [17]. The SPOR Evidence Alliance focuses on promoting evidence-informed health policy, practice, and service to ensure that decision-makers have access to up-to-date scientific evidence. They provide an online form for those seeking specific evidence to inform decisions related to health policy, practice, and service for COVID-19 to facilitate the acquisition of relevant, accurate, and timely data [18]. Several provincial health authorities have also engaged with companies that provide assistance with COVID-19 case contact monitoring; however, information on these was not yet made public or readily available.

#### The United Kingdom

In the United Kingdom, many common in-person medical communications have transitioned into virtual communications. For example, testing centers are booking COVID-19 tests online, and care homes are arranging all their regular resident and staff testing virtually. For those who are self-isolating, the national health service (NHS) has provided information to local authority helplines if self-isolating individuals require practical or social support, support for someone a patient cares for, and financial support. The NHS is also supporting a virtual clinic service powered by medio.link, which is video link–based [19]. They are working with Barts Health NHS Trust to accomplish their goal of forming a national network of sites providing virtual care. The video consultations have been able to be adapted for a variety of settings, including across the entire prison estate. Additionally, video-observed therapy is being substituted for in-person, directly observed therapy to ensure patients with tuberculosis take their medication [20,21]. Digital risk assessment has been established where the analysis of primary care has been linked to mortality data, permitting the risk of death during the first wave to be automatically calculated in providing appropriate advice to guide the patients. The United Kingdom also has a locally developed app called Babylon Health, which is paid for by the client in order to consult virtually with medical staff affiliated with Babylon Health, not necessarily their primary care provider. Additionally, various symptom checker apps such as COVID Symptom Study, Ask NHS, and Symptomate are available for the public’s use to assess whether they could be exhibiting COVID-19 symptoms. There is also Zoe Symptom Tracker, an app that allows for syndromic surveillance and assessment of symptom profiles.

With regards to other tracking options, the United Kingdom is also working on an app to track COVID-19 patients, which has completed trials on the Isle of Wight. Initially, the release of the app saw low download rates, with The Guardian reporting only 10% of the population in both England and Wales [22]. In addition to using Bluetooth technology similar to Canada’s contact tracing app, it enables QR code scanning at venues so that if an individual who recently attended the venue reports a COVID-19–positive test result, other patrons of the venue are automatically notified. In addition to the app, the NHS is providing NHS Test and Trace, a free service that helps trace recent contacts of those testing positive for COVID-19 and notifying them, advising them to self-quarantine. There has also been an increase in digital surveillance throughout the United Kingdom, especially with venues and large employers. For example, daily surveys of hostel managers were performed to facilitate rapid testing and University College London is launching a program, Connect to Protect. This program aids people to report symptoms and positive tests to identify clusters in class, buildings, and residences [23].

#### Rwanda

Rwanda utilizes several existing virtual and mHealth solutions in its ecosystem. To assist in monitoring and supporting patients (cases and contacts) directly under home-based isolation and quarantine, they rapidly deployed the WelTel mHealth platform, which they had previously been using to support patients attending HIV clinics for adherence support. WelTel is an integrated virtual care and 2-way patient engagement digital health intervention that acts as a hub for HCPs to communicate with their patients. As it is primarily used for SMS-based text messaging, patients do not need a smartphone or internet access in order to communicate with their HCPs. WelTel was launched in the Rwanda national emergency operations center for the COVID-19 response in mid-March, within a week of identifying the opportunity of using SMS to reach patients, as previous technologies to reach out to Ebola contacts that were online had limited uptake due to accessibility of the internet. Currently, the WelTel platform is being used for virtual home-based care of COVID-19 patients who are asymptomatic. Above 80% of people who tested COVID-19 positive in Rwanda are asymptomatic. This platform enables the daily follow-up of patients at their home as well as their contacts. The program was rolled out, and Rwandan COVID-19 response teams were trained. The WelTel platform offers several options to interact with the patient through SMS chatting, email, or video call. In addition, at the beginning of the outbreak, WelTel was used to communicate COVID-19 testing results, mainly with those who tested negative. Furthermore, Rwanda uses the DHIS2 (District Health Information Software 2) tracker to record everyone having a COVID-19 test, and it is linked to laboratory systems that push results to the patient’s mobile number and email. The data gathered through Weltel are stored at the Rwanda National Data Center. Rwanda has implemented an additional mHealth technology to help its citizens access HCPs. For example, robots have been implemented in health care centers to collect important patient information including temperature screenings and vital readings, to deliver video messages, and to instruct people to put on a mask. Most importantly, robots play a key role in reducing the exposure time of health care professionals with COVID-19 patients [24]. Two robots have even been deployed at the Kigali International Airport for screening and informing security of issues [25]. The Government of Rwanda has recently (February 2020) signed a 10-year partnership with Babylon’s Rwanda-focused virtual care subsidiary, Babyl. Appointments are being paid for by the government’s health insurance scheme. The service includes physician consults, prescriptions, and lab test codes. There are currently 2 million users. Instructions for Rwandans who might suspect they have COVID-19 are advised to call 114, dial *114# for automated screening, send an email, or send a WhatsApp message [26]. WelTel is also being expanded for remote virtual care for HIV and maternal child health due to access issues under COVID-19.

#### Kenya

Kenya has a few different resources for virtual care using mHealth technology. mDaktari is providing virtual access to primary care via teleconsulting. Patients can access virtual care either through the app or web account, choose an available physician from a directory, or consult an expert using online video or voice calling [27]. A recent Kenyan start-up, mTIBU, focuses on connecting patients to health care affordably without them having to leave home by using the mTIBU mobile app. They offer a variety of medical care including COVID-19 tests, medical consultation, and sample collection, all within the comfort of a patient’s home [28]. In Samburu County, a rural area of northern Kenya, the cStock approach is being used by combining mobile technology, user-friendly dashboards, and quality improvement teams. It is customized for reporting and resupplying health products managed at the community level. Specifically, it is being implemented by the Samburu County Department of Health to supply COVID-19 primary protective gear with support by John Snow International. WelTel is also being used in Samburu County to assist HIV+ patient care and maternal, neonatal, and child health care. COVID-19 health care data are being reported using the DHIS2 tool. Safaricom is being used to send free text messages to educate the community on COVID-19. Health care workers are provided free courses on COVID-19, accessible via smartphones or computers.

**Figure 1:**
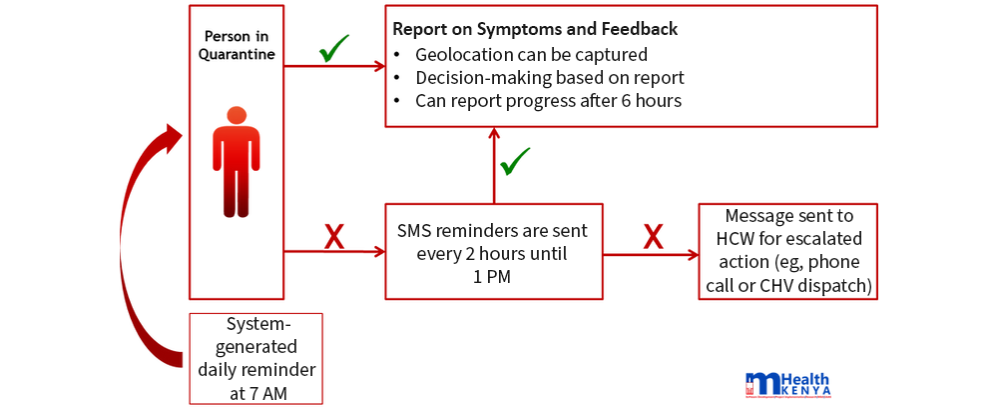
Schematic of the basic functionality of Jitenge on how it works with individuals in quarantine. Image used with permission provided by mHealth Kenya.

There is a national mobile technology–focused group, called mHealth Kenya, which has developed a National Emergency System meant to capture, report, and view emerging epidemics. During COVID-19, the app Jitenge was developed, which allows registered users, either through self-registration or by Ministry of Health officials, to receive daily reminders and prompts to report their health status. The Jitenge system (Figure 1) manages and monitors home-based care management, self-quarantine for contacts, postisolation follow-up, and the monitoring of long-distance truck drivers [29].

With regards to contact tracing COVID-19 patients, an app is available called KoviTrace. It was developed at Mount Kenya University, and it uses a geo-sensing technology to track a patient’s location over a 14-day period when they test positive for COVID-19. If an individual is in close proximity to a COVID-19–positive patient, SMS messages are sent with instructions, and it contacts a COVID-19 response team, depending on the contact’s location [30]. Crowdsourcing movement in Red Zones using social media is also being implemented to aid in contact tracing.

## Discussion

With an urgent, international emphasis on minimizing in-person communication, the development and implementation of mHealth technologies has undoubtedly increased in response to the current COVID-19 pandemic. It is evident that a wide variety of mHealth and virtual care tools with parallel functionality are being used in different sectors of health care throughout North America, Europe, and Africa. The key advantage that these technologies have is that they facilitate both the dissemination and collection of important health information while maintaining safe physical distancing. The most consistently used method of patient care involves teleconferencing and videoconferencing where patients are able to directly communicate with their health care practitioner. A few major players being used internationally include WelTel and Babylon Health, both emphasizing the importance of expedient and accurate delivery of health information and care.

Virtual care tools that facilitate direct communication with HCPs provide a secure way to disseminate or renew prescriptions and provide referrals, common patient needs that are easily addressed without the need for an in-person visit. There is also the added advantage of conversations between patients and HCPs being accessible by either party, especially in text message–based interactions where having access to prior conversations can help with care by verifying details. Traditionally, access to a patient’s health information is held by their HCP; however, being able to refer to conversations is not only useful for the patients, but also for other HCPs to understand what was discussed with the patient. This feature is especially useful for patients communicating with a clinic where different staff are responsible for monitoring a patient’s care.

Public health is also of primary concern, and all the countries discussed in this review are investing resources into national case contact tracing technologies to be able to track COVID-19 infections among their populations. A combination of locally developed smartphone apps and government-implemented technology is being used in order to understand, and hopefully mitigate, the spread of COVID-19. Although a variety of different technologies is being used, there is an emphasis to ensure that those within the vicinity of potentially COVID-19–positive patients are notified and asked to self-isolate. Encouraging self-isolation is occurring at an international level, although regular contact with those in self-isolation varies among the countries we looked at. In general, contact tracing apps can provide more information than just COVID-19–proximity notifications. Data are generated whenever a COVID-19 notification is presented on an individual’s phone, including how many individuals are in the proximity of a positive case as well as when the notification occurred. This information can be useful in figuring out trends of when higher instances of potential exposures are happening most often. Due to the nature of most apps, this information is available immediately and can be used to provide useful information for health authorities to inform decision-makers about how best to mitigate transmission. Moreover, depending on the location privacy of contact-tracing apps, it could be possible to identify zones where transmissions frequently occur. This information can also be stored for future reference.

The type of technology used to manage COVID-19 between the high-income and low-middle income countries was similar; however, Kenya and Rwanda had a more comprehensive approach when using novel technology. Studies evaluating the technical efficiency and overall performance of national COVID-19 management have consistently criticized the United Kingdom and Canada as underperforming despite having access to exceptional resources [31-33]. In fact, the Lowy Institute assessed the performance during the first wave of 98 countries by evaluating various parameters including confirmed cases and deaths per million people and confirmed the cases as a proportion of tests [32]. Their evaluation ranked Rwanda as 6th, Kenya as 48th, Canada as 61st, and the United Kingdom as 66th, indicating that many low-middle income countries were more effective in managing the initial impact of the COVID-19 pandemic than high-income countries. It is challenging to determine exactly how Rwanda and Kenya’s mHealth and virtual care interventions played a role in providing efficient COVID-19 management, but their early development, adoption, and utilization of these tools likely contributed to their effective response to the first wave of the pandemic. In fact, a health care worker based in the United Kingdom responded to an international evaluation assessing COVID-19 management strategies stating that “There was a national plan but it was not effectively put into action” [33].

The implementation and utilization of mHealth and virtual care interventions have grown rapidly during the COVID-19 pandemic as a result of maintaining social distancing measures while providing much needed health care. Although it is not yet known which interventions are the most effective, it is evident that there is consistency with direct virtual health care provider and patient interactions as well as with case contact tracing to notify individuals in efforts to prevent the spread of COVID-19. Studies evaluating patients’ satisfaction with virtual care and mHealth technologies, both before and during COVID-19, have been overwhelmingly positive, strengthening the likelihood that these interventions will become integrated as a regular component of patient care [34-38]. A significant observation of these studies was that patient care was comparable between in-person and virtual consultations; however, the increased convenience for both the HCP and patient and the decreased overall cost for virtual consultations were consistently noted as incentives for adopting mHealth interventions [34,36-38]. The staggering international increase in the adoption of mHealth and virtual care interventions facilitates researching and comparing their efficacy, paving the way to incorporating them into everyday health care globally.

## Appendix

Multimedia Appendix 1 Summary of online resources available to the public to access virtual care and public health resources in Canada, the United Kingdom, Rwanda, and Kenya, during the initial wave of COVID-19.

## Abbreviations

DHIS: District Health Information Software
HCP: health care provider
mHealth: mobile health
NHS: national health service
