# Supplementary material for: Digital mHealth and Virtual Care Use During COVID-19 in 4 Countries: Rapid Landscape Review
Source: JMIR Form Res. 2022 Nov 30;6(11):e26041. doi: 10.2196/26041 (PMC9714961; doi:10.2196/26041)
Supplement: Multimedia Appendix 1 [file formative_v6i11e26041_app1.docx]

Appendix

Appendix Table 1: Summary of Canadian provincial website links to access virtual care during COVID-19 pandemic

| PROVINCE | TOOLKIT/HEALTH AUTHORITY WEBSITE |
| --- | --- |
| British Columbia | <https://www.doctorsofbc.ca/sites/default/files/dto_virtual_care_toolkit.pdf> |
| Alberta | <https://www.albertadoctors.org/leaders-partners/ehealth/virtual-care#toolkit> |
| Saskatchewan | <https://www.sma.sk.ca/kaizen/content/files/SMA_Virtual%20Care%20Quick%20Start%20Guide%20April%2009%202020.pdf> |
| Manitoba | <https://doctorsmanitoba.ca/wp-content/uploads/2020/03/Video-Visit-Apps.pdf> |
| Ontario | <http://www.ontarc.com/covid-19/virtual-visits-toolkit.html> |
| Québec | <https://www.cnesst.gouv.qc.ca/salle-de-presse/covid-19-info-en/Pages/toolkit.aspx> |
| Newfoundland & Labrador | <http://www.nlma.nl.ca/Page/COVID19/Virtual-Care> |
| Nova Scotia | <http://www.nshealth.ca/virtual-care> |
| New Brunswick | <https://www.evisitnb.ca/> |
| Prince Edward Island | <https://www.princeedwardisland.ca/en/information/health-pei/virtual-care-during-covid-19> |
| Yukon Territories | <https://yukon.ca/en/health-and-wellness/care-services/how-check-your-telehealth-appointment-doctor> |
| Northwest Territories | <https://www.nthssa.ca/en/services/virtual-care> |
| Nunavut | <https://www.gov.nu.ca/health/information/telehealth> |

Appendix Table 2: Summary of technology and its primary purpose used within each country discussed.

| COUNTRY | PRIMARY PURPOSE | TECHNOLOGY | LINK |
| --- | --- | --- | --- |
| *Canada* | Public Health* | COVID Alert – Canada (Ontario) | <https://www.canada.ca/en/public-health/services/diseases/coronavirus-disease-covid-19/covid-alert.html> |
|  |  | ABTraceTogether - Alberta | <https://www.alberta.ca/ab-trace-together.aspx#download> |
|  |  | Mila - Quebec | <https://mila.quebec/en/a-smart-and-ethical-contact-tracing-app-to-fight-covid-19/> |
| *UK* | Virtual Care | Virtual Clinic - NHS | <https://medio.link/virtual-clinic/> |
|  |  | Babylon Health (app – paid for by patient) | <https://www.babylonhealth.com/> |
|  | Public Health | UK Track & Trace App | <https://covid19.nhs.uk/> |
| *Rwanda* | Public Health &  Virtual Care | WelTel | <https://www.weltel.rbc.gov.rw/> |
|  | Virtual Care | Babyl – of Babylon | <http://www.babyl.rw/> |
| *Kenya* | Virtual Care | ConnectMed | https://hmis.moh.gov.rw/covid19/dhis-web-commons/security/login.action |
|  | Public Health | KoviTrace | <https://itweb.africa/content/mQwkoq6PgWk73r9A> |
